# Prognostic Factors Determining Morbidity and Mortality in Organophosphate Poisoning

**DOI:** 10.12669/pjms.333.12395

**Published:** 2017

**Authors:** Ayca Acikalin, Nezihat Rana Dişel, Selcuk Matyar, Ahmet Sebe, Zeynep Kekec, Yuksel Gokel, Emre Karakoc

**Affiliations:** 1 Ayca Acikalin, Cukurova University Faculty of Medicine, Department of Emergency Medicine. Cukurova University Faculty of Medicine, Adana, Turkey; 2 Nezihat Rana Dişel, Cukurova University Faculty of Medicine, Department of Emergency Medicine. Cukurova University Faculty of Medicine, Adana, Turkey; 3 Selcuk Matyar, Biochemistry Division of Laboratory. Cukurova University Faculty of Medicine, Adana, Turkey; 4 Ahmet Sebe, Cukurova University Faculty of Medicine, Department of Emergency Medicine. Cukurova University Faculty of Medicine, Adana, Turkey; 5 Zeynep Kekec, Cukurova University Faculty of Medicine, Department of Emergency Medicine. Cukurova University Faculty of Medicine, Adana, Turkey; 6 Yuksel Gokel, Cukurova University Faculty of Medicine, Department of Emergency Medicine. Cukurova University Faculty of Medicine, Adana, Turkey; 7 Emre Karakoc, Department of Internal Medicine, Intensive Care Unit. Cukurova University Faculty of Medicine, Adana, Turkey

**Keywords:** Mortality, Organophosphates, Prognosis, Therapeutic plasma exchange

## Abstract

**Objective:**

Our aim in this retrospective study was to determine the factors affecting poor prognosis and mortality of organophosphate (OP) poisoning by reviewing patient data. We also reviewed present knowledge to make conclusions on certain longstanding debates in light of the literature.

**Methods:**

In this retrospective descriptive study, patients who were admitted to and hospitalized in the emergency department (ED) or intensive care unit (ICU) of a university hospital with the diagnosis of OP poisoning between December 2010 and December 2015 were evaluated. All the data were obtained from electronic and manual patient files. A total of 80 patients were included in the study.

**Results:**

The mean age of the study patients was 32.4±15.0 (13-94). Forty-nine (61.2%) patients were female. Twenty-two (27.5%) patients were seriously poisoned and needed mechanical ventilation (MV) support. Low pseudocholinesterase (PChE), high creatinine (Cr), low Glasgow Coma Scale (GCS) scores and long hospitalization durations were all found to be poor prognostics in MV patients. Low PChE and high Cr levels were found to be independent predictors of the hospitalization duration and high Cr was found to be an independent predictor of the intubation duration of MV patients in regression analyses. Ten (45.5%) of the MV patients were unresponsive to medical treatment and Therapeutic plasma exchange (TPE) was performed. Seven patients were discharged healthy. Three patients with low PChE levels and comorbidities died.

**Conclusions:**

Prolongation of respiratory depression necessitating MV support, comorbidities, long hospital stay, elevated creatinine, low GCS scores and low PcHE levels without regeneration in the first 48 hours of admission are all found to be poor prognostic factors for organophosphate (OP) poisoning.

## INTRODUCTION

Organophosphate compounds (OPs) are widely used as insecticides all over the world. These highly toxic compounds may cause mortal poisoning in humans. Lipophilic properties of these compounds make them absorbable quickly via the skin. OPs are also used as chemical weapons due to high toxicity when inhaled.^1^

Toxic exposure to OPs may be accidental or intentional for committing suicide in Turkey.^2^ OPs cause cholinergic syndrome, which may result in death due to inhibition of the enzymes acetylcholinesterase (AChE) and butyrylcholinesterase [(BuChE) or pseudocholinesterase (PChE)]. The mortality rate of OP poisoning remains as high as 10-20% despite widely available antidotes used in treatment.^3^ Fatalities are related to cholinergic syndrome in acute phases and intermediate syndrome (IS) in late-onset cases. Thus, indicators predicting mortality and morbidity are being investigated. In literature, numerous scoring systems were used to assess patients with poor prognoses, such as the Glasgow Coma Scale (GCS), the Acute Physiology and Chronic Health Evaluation (APACHE-II), the Simplified Acute Physiology Score (SAPS),^4^ Body Mass Index (BMI),^5^ plasma cholinesterase (PChE) levels, biochemical and inflammatory response markers and red cell distribution width (RDW).^6^ However, debates continue over indicators of poor prognosis and mortality. It is important to identify patients with poor prognosis and high mortality in order to refer them to toxicology centers to test the feasibility of extracorporeal elimination methods in the treatment. Our aim in this study was to determine the factors affecting poor prognosis and mortality by reviewing data of our patients. We also reviewed present knowledge to make conclusions on debates in the literature.

## METHODS

This is a retrospective descriptive study of OP poisoning patients in a period of five years. The study was approved by the ethical committee of the institution. Patients included in the study had been admitted to and hospitalized in the emergency department (ED) or intensive care unit (ICU) of a university hospital with the diagnosis of OP poisoning between December 2010 and December 2015. A total of 107 patients were evaluated. All data were obtained from electronic and manual patient files. Patients with hematocrit levels below 26%, aged under 14 years old and whose clinical data were absent were excluded (n=27).

A standard data sheet was used to record age, gender, method of poisoning (accidental oral intake, occupational exposure in farming, committing suicide, etc.), GCS scores, need for mechanical ventilation (MV), number of days on MV therapy, hospitalization duration, method for extracorporeal elimination and the outcomes of the patients. Besides demographic data and laboratory results, the causes of death for fatalities were also recorded.

### Statistical Analyses

Analyses were performed by the SPSS 15.0 statistical software pack. Parametric demographic data and laboratory findings were evaluated by the Student’s t test and categorical data by the Chi-square test and Fisher’s exact tests. Measurement methods were compared by correlation analysis. Linear regression analyses were performed to study the relation between variables. Statistical significance was set at a value of p<0.05.

## RESULTS

Eighty patients were included in the study through record. The mean age was 32.4±15.0 (13-94). Forty-nine (61.2%) were female. Exposure patterns were as follows: oral intake for suicide attempts in 60 patients (75%), occupational exposure during spraying for pest control in the field in 11 patients (13.8%), accidental oral intake along with food in five patients (6.2%), and wiping on head as a treatment for pediculosis in four patients (5%). Patients’ characteristics are summarized in Table-I..

**Table-I:** The characteristics of the patients.

*Parameters*	*Patients’ Results Mean ± SD (min-max)*
Age (years)	32.4±15.0 (14-94)
Female/Male (n, %)	49/31 (61.2/38.8)
GCS on admission	12.9±3.2 (6-15)
Intubation and MV (n, %)	22 (27.5)
Duration of intubation of the discharged patients (days)	0.86 ±1.97 (0-10)
Duration of hospitalization of the discharged patients (days)	4.7±3.2 (1-16)
TPE (n, %)	10 (12.5)
Exitus (n, %)	3 (3.8)

Twenty-two (27.5%) of the patients needed MV support, The comparison of the patients with MV support to those without is summarized in Table-II.

**Fig.1:**
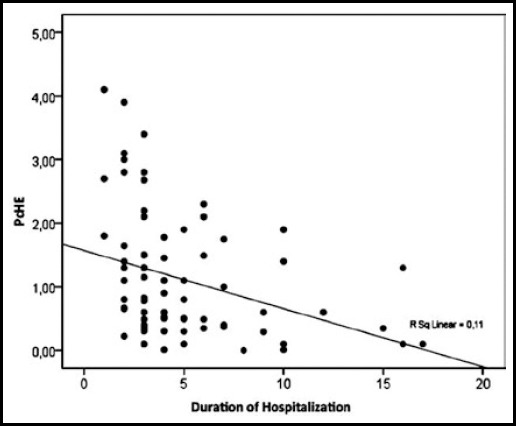
Regression analysis for hospitalization duration and PChE.

**Fig.2:**
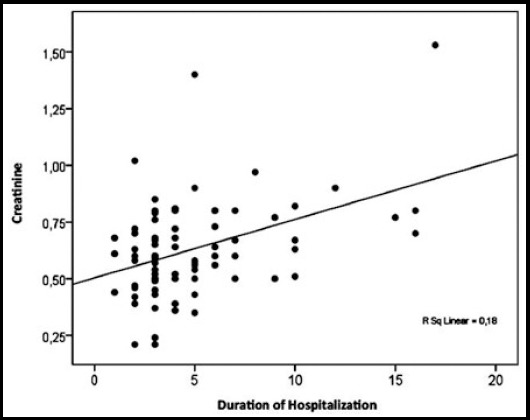
Regression analysis for hospitalization duration and Cr.

The PChE and Cr levels and hospitalization durations of the patients with MV support were statistically different from those without (Table-II). There was a positive correlation between hospitalization duration and Cr (p=0.016, r=0.274) where a negative correlation was found for PChE (p=0.008, r=-0.301) (Fig.1 and 2). In regression analyses, the PChE and Cr levels were found to be independent predictors of hospitalization duration (Table-III). In addition, the Cr level was found to be an independent predictor of intubation duration (p=0.031).

**Table-II:** The comparison of the patients (MV support versus non-MV).

	*MV(-) (n=58)*	*MV(+) (n=22)*	*p*
Age (years)	31.0±14.4	36.0±16.1	0.208
Female/male	38/20	11/11	0.203
Erythrocyte (10^6/μL.)	4.7±0.5	4.4±0.7	0.111
Hemoglobin (g/L)	13.2±1.4	12.9±2.1	0.505
Hematocrit (%)	38.9±3.9	37.7±5.8	0.348
MCV (fL)	84.5±6.7	85.7±6.3	0.460
White blood cell(x1.000/μl)	13.1±5.1	12.5±4.1	0.607
Platelet(103/ μL)	261.3±78.8	252.9±81.3	0.678
RDW(%)	14.6±2.1	14.0±1.0	0.080
BUN(mg/dl)	11.4±3.9	14.2±8.5	0.144
Fasting glucose(mg/dL)	124.4±39.4	177.8±103.8	0.028*
Creatinine(mg/dL)	0.59±0.16	0.75±0.29	0.018*
ALT(U/L)	23.1±34.8	29.6±30.5	0.415
AST(U/L)	25.9±17.9	32.3±19.9	0.198
PKE(KU/L)	1.29±1.01	0.63±0.63	0.001*
GCS	14.6±0.8	8.1±2.1	0.001*
TPE(n,%)	0(0.0)	10(45.5)	0.001*
Duration of hospitalization(day)	3.5±1.8	8.4±4.0	0.001*
Exitus (n, %)	0(0.0)	3(13.6)	0.019*

* normal level for PChE is 4,9-11,9 KU/L

**Table-III:** Regression analysis for hospitalization duration.

	*Odds ratio*	*SE*	*95%Cl*	*P*
Constant	2.937	1.317	(0.311) - (5.563)	0.029
PKE	-1.096	0.370	(-1.834) - (-0.358)	0.004
Cr	4.908	1.936	(1.046) - (8.771)	0.013

TPE was performed on 10(45.5%) patients who were unresponsive to antidotal and supportive treatment and under continuous MV support due to respiratory failure. Three of these patients died and detailed data of their cases are summarized in Table-IV.

**Table-IV:** Demographic and laboratory results of the patients who died.

*Parameter*	*Patient 1*	*Patient 2*	*Patient 3*
Age	62	51	67
Sex	Female	Male	Male
Method	Suicide	Suicide	Accidental
Agent	Diazinon	Diazinon	Diazinon
Comorbidity	HT, DM, Obesity	HT, DM, Obesity, CAD	HT, DM, CAD
Initial PChE	0,1KU/L	<0.1KU/L	<0.1KU/L
PChE at 48^th^ hour	0.1KU/L	<0.1KU/L	<0.1KU/L
Respiratory situation	Delayed admittance at 12^th^ hour of ingestion, Immediate Intubation and MV support	Intubation at 24^th^ hour, MV support, Intermediate Syndrome	Intubation at 3^rd^ day, MV support, Intermediate Syndrome
Number of MV days	17	7	92
Hospitalization time	Exitus at 17^th^ day	Exitus at 8^th^ day	Exitus at 95^th^ day
Complications	Non-STMI, Pulmonary Thromboembolism	Non-STMI, Atrial Fibrillation	Non-STMI, UGIB, Ventilator-Related Pneumonia, Septic Shock

***Abbreviations:*** CAD: Coronary Arterial Disease; DM: Diabetes Mellitus; HT: Hypertension; MV: Mechanical Ventilation; Non-STMI: Non ST elevation myocardial infarction; PChE: Pseudocholinesterase; UGIB: Upper gastrointestinal bleeding.

## DISCUSSION

OP poisonings are not rare in Turkey and are usually due to accidental oral intake, exposure during spraying of fields, mistreatment for pediculosis and more often, suicidal intention. Despite having antidotes, OPs cause serious toxicity, even death. Several factors affect mortality in OP poisoning. Acute cholinergic crisis and respiratory failure, such as Intermediate Syndrome are frequently related to death.^7^ Mortality rates are reported to be as high as 10-20% in the literature.^1^,^6^-^9^ In our study, the mortality rate was 3.8% (n=80/3) in all patients and 13.6% (n=22/3) in the patients who needed MV support.

Patients requiring mechanical ventilation due to respiratory depression usually necessitate prolonged MV support. Excessive bronchorre, fatigue of respiratory muscles, central nervous system toxicity causing hypopnea, aspiration of the gastric contents or even aspiration of active charcoal in the patients who do not have secure airways are the causes of early onset pneumonia in OP patients. Mechanically ventilated patiens face up with endotracheal tube and/or bronchial obstruction due to thick sputum secondary to atropine treatment. Patients need frequent deep tracheal suction which cause contamination. These mucus plugs also cause atelectasis. Sedoanalgesic side effects, intermediate syndrome, ventilatory related pneumonia, barotrauma, oxygen toxicity, pulmonary embolism and cardiac complications are all other causes of morbidity and mortality.

Many studies have investigated the need for MV support in OP poisoning cases. The ratio of patients who needed MV support was reported to be 34.2%(26/76 patients), 43.8% (49/112 patients), 20.8%(15/72 patients) and 24%(90/376 patients) in the studies of Muley et al., Lee et al., Dundar et al., and Eddleston et al. respectively.^5^,^7^,^8^,^10^ These studies reported that MV was a factor affecting hospitalization duration and long hospitalization durations were related to high mortality.^5^,^7^,^8^,^10^ In our study, 22 of the 80 (27.5%) patients needed MV support, and hospitalization durations and mortality rates for these patients were higher than in those who did not need MV (Table-II).

Another prognostic criterion which had been studied previously was severity scoring systems. APACHE-II, SAPS-II, GCS, and PSS (poisoning severity score) were all studied. APACHE-II, GCS, and PSS scoring systems were found to be correlated to mortality rates.^11^,^12^ We found that patients with low GCS were hospitalized longer (p<0.001, r=-0.723). With regression analyses for GCS, PChE and Cr were found to be independent predictive parameters. Low GCS are seen frequently in severe OP poisoning cases. Direct cerebral toxicity of the agents and hypoperfusion or hypoxemia due to respiratory failure usually causes low GCS scores. Elevated Cr levels may be the reason for multiorgan failure due to the agent itself; they are rarely due to acute toxicity or secondary concomitant diseases of the patients, such as DM and HT. Comorbidities complicate and obstruct treatment of OP poisoning. Atropine may cause arrhythmias or acute coronary syndromes in patients with coronary heart disease, DM and HT due to an increase in oxygen consumption of myocardium due to tachycardia. Elongation of the QT interval in electrocardiogram is said to be a mortality indicator and myocardial ischemia and ventricular arrhythmias affect mortality^13^ All three deceased patients had DM and HT on admission. Non-STMI presented in all, and one had atrial fibrillation with rapid ventricular response during hospitalization. Atropine infusion had to be tapered but cholinergic symptoms emerged immediately; this was the most difficult and challenging period of the treatment.

Measurements of plasma BuChE (PChE) and erythrocyte AChE levels are used in the diagnosis and management of OP poisoning. Detection of PChE levels is preferred because of being cheaper and easier.^14^ The severity of the poisoning and decrease in PChE levels are correlated in many cases, though some studies indicate that this decrease is diagnostic but not correlated to clinical severity of poisoning.^15^ Many other studies report correlation of low PChE levels with mortality rate, poor prognoses and long hospitalization duration.^16^ In a retrospective study conducted by Gazzi et al., 606 patients over 30 years were evaluated and low PChE levels were reported to be an indicator of long hospitalization duration.^17^ Moon et al. reported that erythrocyte AChE levels in the first 24 hours may predict MV duration but was not a useful indicator to assess weaning.^18^ Our findings revealed that PChE levels of the patients who needed MV were extremely low (p=0.001). Furthermore, patients having long hospitalization duration (p=0.008, r=-0.301) and low GCS scores (p=0.003, r=0.330) had significantly low PChE levels. Regression analyses showed independent predictors for hospitalization duration are PChE and Cr levels, and Cr levels are also a predictor for intubation duration.

The deceased patients in our study had decreased PChE levels. No regeneration occurred in the first 48 hours and these levels stayed low during hospitalization. Severity of the poisoning may be correlated to low PChE levels because patients having extremely low PChE levels had low GCS scores and long hospitalization durations. No regeneration of the enzyme was detected in the deceased patients. This may be explained by the aging of the enzyme-OP bond. This is called “aging” and the aging time of each compound differs from minutes to days.^9^ All three deceased patients had ingested Basudin 60 M® (Diazinon) and all had decreased initial PChE levels as low as <0.1 KU/L. Despite resolution of cholinergic symptoms by atropine and 2-PAM treatments, respiratory muscle weakness and respiratory failure persisted, requiring long MV support. We observed that aging was accelerated in diazinon poisoning and while muscarinic symptoms were treated, nicotinic symptoms like muscle weakness lasted longer. Complications during MV added comorbidities and led to death in these patients.

One patient was diagnosed with ventilator-related pneumonia and septic shock, and another one had pulmonary thromboembolism. In Sun et al.’s retrospective study, 81 discharged patients were compared to 11 deaths and pneumonia was found to be the most frequent cause of death.^19^

Utilization of extracorporeal elimination methods are suggested in patients who have no clinical improvement in respiratory failure (intermediate syndrome) and cholinergic manifestations despite antidotal and supportive treatment.^20^ There are studies in the literature concluding that elevation in plasma cholinesterase levels and improvement in respiratory functions are achieved by TPE.^20^,^21^ Our study’s results support the effectiveness of TPE in the patients under prolonged MV support. Seven of 10 (45.5%) patients who were performed TPE were discharged healthy in our study.

### Limitations

This is a retrospective study. Lack of data in the patient files, difficulty in standardization of patient characteristics (age, gender, treatments performed before transfer to our clinic) are some of the limitations. The age interval was wide (13-94).

## CONCLUSION

Complications faced during MV support in OP poisoning patients and accompanying comorbidities are found to cause mortality. Despite effective antidotal theraphies, being unable to cope with respiratory depression, low GCS scores, prolonged MV support and hospitalization, elevated creatinine, low initial PChE levels, and persistence of low enzymes at the 48th hour of admission are all found to be indicators of poor prognosis.
